# Psychometric properties of the international otcome inventory for hearing AIDS

**DOI:** 10.1590/S1808-86942010000100014

**Published:** 2015-10-17

**Authors:** Marisa Gasparin, Isabela Hoffmeister Menegotto, Cristine Santos da Cunha

**Affiliations:** 1 Graduate student, Intensive Care - Integrated Health Residency Program, GHC, Speech and Hearing Therapist. Intensive Care Resident Physician, Integrated Health Residency Program, Grupo Hospitalar Conceição; 2 PhD on Speech and Hearing Therapy, Human Communication Disorders, Adjunct Professor, Speech and Hearing Therapy Program, Brazilian Lutheran University, Clinical Speech and Hearing Therapist, Medicina Diagnóstica Mãe de Deus Center; 3 Speech and Hearing Therapist

**Keywords:** hearing aids, questionnaires, rehabilitation

## Abstract

It is paramount to assess the psychometric properties of self-assessment tools in order to check the tests' reliability and validity, also to enable proper outcome interpretation.

**Aim:**

to check the psychometric properties of the IOI-HA (International Outcome Inventory for Hearing Aids) in its Portuguese version, called QI-AASI (International Questionnaire - Individual Sound Amplification Device), in terms of internal uniformity, correlation between the items and reproducibility. Study design: descriptive, observational and cross-sectional.

**Materials and Methods:**

the questionnaire was deployed to 53 hearing aid users, 34 females and 19 males, with ages between 19 and 92 years - from incomplete basic education to complete higher education, encompassing subjects with monoaural and binaural sound amplification.

**Results:**

the QI-AASI had a Cronbach Alpha of 0.69. In the correlation among the items, there were numerous significant correlations. The instrument was properly reproducible, except for item # 6, which presented a significant difference in comparing test and the retest.

**Conclusions:**

the QI-AASI is suggested in the rehabilitation process of users of hearing aides; nonetheless, the questionnaire can be difficult for subjects with low social and economic status when self-employed.

## INTRODUCTION

Hearing is one of the most important senses, as it is the basis for language acquisition and development. In today's world, communication is a fundamental aspect in the insertion of individuals into social life; communication disorders may thus introduce a series of burdens in the life of individuals suffering from them.

Hearing aids are sound amplification devices used to facilitate communication involving people with hearing loss. A well-fitted device improves the user's quality-of-life, bringing comfort, a sense of well-being and minimizing the barriers posed by hearing loss.

Various questionnaires have been developed with the purpose of assessing the benefits yielded by hearing aids in daily life situations; among them is the IOI-HA (International Outcome Inventory for Hearing Aids). This questionnaire has been translated into 21 languages, and in Portuguese it is known as the QI-AASI (Questionário Internacional - Aparelho de Amplificação Sonora Individual). This assessment tool is made up by seven questions used to subjectively look into the results yielded by the hearing aids under the following parameters: 1 - time for which hearing aids have been used; 2 - benefit; 3 - residual limitation in daily life activities; 4 - satisfaction; 5 - residual restrictions to participation; 6- impact on other people; 7 - quality of life. Five graded responses can be given to each question, ranging from poor performance (1) to best performance (5)^1^.

Tools aimed at measuring the degree of benefit perceived by hearing aid users must be reliable. Therefore, to analyze the psychometric properties of the IOI-HA is of paramount importance in order to test how reliable and valid the intended measurements are and for its results to be accurately interpreted. The psychometric parameters of the IOI-HA have been described for other languages^2^, ^3^, ^4^ and research findings support the use of this tool in the rehabilitation process of hearing aid users.

Therefore, this papers aims to analyze the psychometric properties of the Portuguese version of the IOI-HA.

## MATERIALS AND METHOD

This study is connected to a research project called 'Reproducibility of questionnaires in Portuguese for validating hearing aid fitting' approved by the Ethics Committee a tour institution under permit 2006-347H.

The questionnaire was applied to hearing aid users based on the following enrollment criteria: unilateral or bilateral hearing aid users; subjects had to be 18 or older; absence of perceptible cognitive disorders; able to respond the questionnaire without the help of third parties; not having undergone speech and hearing therapy and/or changes to the hearing aid between testing and retesting.

Seventy-two subjects were called based on information locally available and through referrals. Two of the subjects refused to participate on the study. After they were informed on the purpose of the study, a visit was scheduled for them to respond to the questionnaire. Seventeen other individuals were excluded from the study as they failed to meet the above mentioned requirements, which left us with a final sample of 53.

On the first visit, all subjects agreed to participate on the study and signed an informed consent term to then fill out a questionnaire containing personal identification data, general information on the type of hearing loss and on the use of hearing aids. They were then given the IOI-HA. The subjects answered the questions by themselves, without the help of third parties, as required by the researcher. Instructions were included in the text preceding each question, and subjects could pick only one answer for each question.

Lesser educated participants and subjects with reading difficulties were read the questionnaire, with questions and possible answers being presented to them in the very same form as they were written. These individuals were grouped separately. In cases of doubt over any of the given questions, the researcher either told the subjects to read the question again or read it to them.

After the questionnaire was applied for the first time, a new appointment was scheduled for retesting, within at least seven days to no more than thirty days. Participants responded the questionnaire again following the same procedure described above, and were not given access to the answers they gave the first time around.

A total of 53 hearing aid users made up the studied sample; 34 (64.2%) were females and 19 (35.8%) were males, with ages ranging between 19 and 92 years. All subjects lived in the State of Rio Grande do Sul, southern Brazil. In terms of formal education, 1.9% of the subjects reported not to have concluded basic schooling; 13.2% concluded basic schooling; 9.4% did not conclude fundamental education; 20.8% concluded fundamental education; 1.9% dropped out of high school; 28.3% concluded high school; 3.8% did not conclude higher education programs; and 20.7% concluded higher education programs.

Binaural use of hearing aids accounted for 24.5% of the sample, while monaural use amounted to 75.5%. Hearing aid types ranged from analog to digital devices, programmable and non-programmable ones. The time for which hearing aids were used by the subjects ranged from 2 months to 32 years; 71.7% of the subjects had been using hearing aids for under 10 years.

The sample was divided into two groups, one of 48 subjects who answered the questions without anyone's help and another with 5 in which the researcher aided them by reading the questions and possible answers.

The answers given to the questions by the subjects were converted into numeric values to allow for statistical analysis. Calculation was done based on the answers given the first time the test was applied. Another two individuals had to be dropped from some of the statistical analysis, as their questionnaires had not been completely answered; we were thus left with a sample of 46 for some of the questions.

Mean values and standard deviations were then analyzed for the entire questionnaire and each individual question. The correlation between each item and the whole test, i.e., the correlation between each item and the total score on the questionnaire was also calculated using Pearson's Correlation Ratio. According to the literature^5^, this is important information as it estimates the discrimination or validity ratio of each item.

The Cronbach Alpha is one of the procedures required to estimate the reliability of a test; there are no rules establishing reference values for ratios, but values above 0.75 are deemed high^5^. Internal consistency was assessed by calculating the Cronbach Alpha of the tool as a whole and for the questionnaire if the item is eliminated. The correlation between items was analyzed using Pearson's Correlation Ratio with a level of significance set at 5%.

Factors are groups of questions covering different areas, i.e., each factor comprehends a group of questions connected to each other. As also done for the questionnaires in English, Dutch, and German^2^, ^3^, ^4^, factorial analysis was done in this study using the varimax rotation method selecting only the factors whose 'autovalues' were greater than 1.

The results obtained from testing-retesting were analyzed and compared using the paired T-test with a level of significance set at 5%. The correlation between pairs was verified again using Pearson's Correlation Ratio with a level of significance set at 5%.

## RESULTS

Table 1 presents the mean values and standard deviations of the answers in the questionnaire in general and for each item after numerical adjustment (analysis after first application - test). Mean values ranged between 3.43 and 4.67. The group that had the questionnaire read to them (n=5) had mean values for each question ranging from 3.80 to 4.80 according to the answers given the first time the test was applied (test). Still on Table 1, the third and fourth columns show the corrected item-total correlation and the Cronbach Alpha of the tool if each item is removed. The Cronbach Alpha for the whole questionnaire (n=46) was of 0.69.

**Table 1:** Mean, standard deviation of answers corrected item-total correlation, and questionnaire Cronbach Alpha IF each item is removed and for the questionnaire as a whole (n=46).

	Mean	SD	Correlation item-total corrigido	Cronbach Alpha if item is removedremovido
Q1	4,67	0,94	0,27	0,67
Q2	3,91	1,03	0,45	0,62
Q3	3,43	1,07	0,45	0,62
Q4	4,26	0,93	0,62	0,58
Q5	3,69	1,26	0,37	0,64
Q6	3,72	1,19	0,17	0,7
Q7	3,91	0,98	0,41	0,63

Total	27,61	4,3		0,69

Legend: Q1: question 1; Q2: question 2; Q3: question 3; Q4: question 4; Q5: question 5; Q6: question 6; Q7: question 7.

The correlation between the items in the questionnaire is presented on Table 2, as described in the materials and method section.

**Table 2:** Correlation between IOI-HA items calculated by Pearson's Correlation Ratio (significant* if p £ 0.05).

		Q1	Q2	Q3	Q4	Q5	Q6	Q7
Q1	Pearson's Correlation	1,000	0,294	0,038	0,373	0,135	0,046	0,231
	p=	(n= 48)	0,045*	0,796	0,009*	0,366	0,757	0,114
Q2	Pearson's Correlation		1,000	0,325	0,635	0,048	-0,103	0,445
	p=	(n= 47)	(n= 47)	0,026*	0,000*	0,753	0,493	0,002*
Q3	Pearson's Correlation			1,000	0,276	0,295	0,243	0,323
	p=	(n= 48)	(n= 47)	(n= 48)	0,058	0,044*	0,096	0,025*
Q4	Pearson's Correlation				1,000	0,332	0,016	0,449
	p=	(n= 48)	(n= 47)	(n= 48)	(n= 48)	0,022*	0,913	0,001*
Q5	Pearson's Correlation					1,000	0,399	0,044
	p=	(n= 47)	(n= 46)	(n= 47)	(n= 47)	(n=47)	0,005*	0,767
Q6	Pearson's Correlation						1,000	0,026
	p=	(n= 48)	(n= 47)	(n= 48)	(n= 48)	(n= 47)	(n= 48)	0,861
Q7	Pearson's Correlation							1,000
	p=	(n= 48)	(n= 47)	(n= 48)	(n= 48)	(n= 47)	(n= 48)	(n= 48)

Legend: Q1: question 1; Q2: question 2; Q3: question 3; Q4: question 4; Q5: question 5; Q6: question 6; Q7: question 7.

The IOI-HA factorial analysis resulted in the extraction of three factors. Factor 1 covered items 2, 3, 4, and 7, connected to benefit, residual limitations to everyday-life activities, satisfaction, and quality of life. Factor 2 was made up by items 5 and 6 on residual restriction to participation, and impacto f hearing loss upon others. Factor was represented only by item 1, on the time for which subjects had been using hearing aids.

Finally, Table 3 contains the testing-retesting reliability of the IOI-HA and the correlation between answers in both test applications.

**Table 3:** Differences between mean values, difference standard deviation, paired T-test (significant* if p £ 0.05), correlation between items in both applications and Pearson's Correlation Ratio (significant* if p £ 0.05).

Question	Difference between mean values SD	Desvio-padrão	p	Correlação	p
(teste-reteste)		p	Correlation	p	0,000**
Q2	0,02	0,45	0,743	0,907	0,000**
Q3	0,00	0,63	1,000	0,836	0,000**
Q4	-0,07	0,44	0,323	0,883	0,000**
Q5	-0,11	0,80	0,359	0,801	0,000**
Q6	-0,35	1,04	0,028*	0,573	0,000**
Q7	-0,02	0,71	0,837	0,723	0,000**

Legend: Q1: question 1; Q2: question 2; Q3: question 3; Q4: question 4; Q5: question 5; Q6: question 6; Q7: question 7.

## DISCUSSION

This study looked into the psychometric properties of the Portuguese version of the IOI-HA in terms of internal consistency, correlation between items and reproducibility.

The mean value for each item, as shown in Table 1, varied between 3.43 and 4.67, the maximum score being 5. This seems to indicate that this group of subjects is relatively satisfied with the hearing aids, as it shows favorable attitudes (above 50% of the total score) towards hearing aids. The literature supports this finding^2^, ^3^, ^4^.

The mean values of the questions for the group of subjects who had the questions read to them ranged between 3.80 and 4.80, a finding quite close to the values seen in the 46-subject sample, indicating that merely having the questions read to may not impact the outcome of the questionnaire.

Table 1 also presents the corrected item-total correlation and the Cronbach Alpha of the questionnaire for each item removed and for the questionnaire as a whole. This correlation should be moderate to high, and any item whose corrected item-total correlation is lower than 0.20 should be removed from the combined count6. In this study, item 6 (impact on others) had a corrected item-total correlation of 0.17. In the analysis looking at the overall Cronbach Alpha IF each item is eliminated, this same question introduced na irregularity, as if it were removed the total Alpha would increase from 0.69 to 0.70. In a study done on the English questionnaire^2^ similar findings were observed for item 5 (residual restriction to participation), and for the German version4 the same was found for item 1 (time of use). If the total Cronbach Alpha increases significantly when an item is removed, there is indication that such item is not consistent enough before all others^2^. This indicates that while the IOI-HA is concise, comprehensive, and accessible by different cultural and social contexts^7^, the same cannot be said in equal terms for its translated versions, even when taking the different sample sizes of the various studies done on the topic.

The questionnaire's internal consistency was measured through the Cronbach Alpha ratio, and a value of 0.69 was found as seen on Table 1. This result is lower than the values observed in the English and German versions, with 0.78 and 0.91 respectively 2,4. The literature indicates that higher Cronbach Alpha ratios imply more reliable tools^5^. The Cronbach Alpha found in this study indicates that the QI-AASI is moderately reliable. It should be noted that in other studies^2^,^4^ the samples were much greater, a fact that increases the probability of achieving a greater Cronbach Alpha.

In the correlation between items presented on Table 2, significant correlations were seen between questions, showing that longer times of use of hearing aids led to greater benefit to the users; the longer subjects used their hearing aids, the more satisfied they were; the greater the benefit, the lesser were the limitations to performing everyday life activities; the greater the benefit the greater the satisfaction; the greater the benefit the greater was quality of life; the fewer the difficulties found with using hearing aids, the less would hearing loss impact the user's ability to perform daily life tasks; the fewer the difficulties found with using hearing aids, the greater was quality of life; the greater the satisfaction, the fewer were the residual restrictions to participation; the greater the satisfaction, the greater was quality of life; and the less hearing loss affects the performance of daily life activities, the lesser is the impact of hearing loss in building relationships with other people.

The factors extracted from the QI-AASI are different from the ones mentioned in the literature^3^, ^4^. Cox^7^ reported that the seven items in the English questionnaire are grouped in two distinct areas, Factor 1 and Factor 2. Factor 1 refers to the joint analysis of items 1 (time of use), 2 (benefit), 4 (satisfaction) and 7 (quality of life), showing how users relate to their hearing aids. Factor 2 is interpreted as the one that reflects the impact of hearing aids in the interactions subjects have with the world, containing items 3 (residual activity limitation), 5 (residual participation restriction) and 6 (impact on others).

In this study the items were grouped in a different manner, as seen in the results. Given that the studied populations are different, it is perfectly possible that questions are grouped differently. In this case, Factor 1 covered items referring to the benefits or limitations that hearing aids produce upon the users. Factor 2 was made up by items related to users and the environment. And Factor 3 was constituted only by the question on time of use of hearing aids unrelated to the other two factors.

The differences between the mean values in testing and retesting are presented on Table 3. The only item in which significant differences were found between testing and retesting mean values was item 6 (impact on others) with a value of −0.35. The negative value indicates that subjects reported more trouble with others the second time they were asked about it; this data is thus interpreted as a worsened perception over the use of hearing aids. No other studies were found to compare against this finding, but one might wonder that after being tested the first time individuals may have reflected and concluded that in spite of the hearing aids their residual hearing loss upsets other people. Another possibility is that subjects may have misunderstood the question. As the questionnaire was applied, we observed that some participants had difficulty understanding some of the questions, a consequence of the low formal schooling profile seen in part of the sample.

Table 3 also presents data on testing-retesting reliability; question 6 (impact on others) had statistically significant differences and showed irregularities in all analyses. As previously described, one of the possible explanations is the difficulty subjects may have had understanding or interpreting this item; or that indeed item 6 presents irregularities in the Portuguese version of the IOI-HA, thus requiring a review. In the study of the Dutch questionnaire3 no statistically significant differences were found between both sessions. Correlation values between items in testing and retesting are shown in the last column, with significant values for all questions. No studies were found to compare this finding against, but such a finding shows that participants had the same behavior in both sessions.

## CONCLUSION

This study presents some preliminary results from the analysis done on the psychometric properties of the Portuguese version of the IOI-HA. We could see that the questionnaire presents moderate levels of internal consistency. Many items are correlated to each other (analysis of first application - test), and the tool can be reproduced adequately, except for item 6 (impact on others), which had statistically significant differences when comparing testing and retesting values.

As also seen in other studies^2^, ^3^, ^4^, the IOI-HA should be used in the rehabilitation process of hearing aid users, although the questionnaire may be difficult to understand by poorly educated subjects when they apply the questionnaire on themselves. It is also believed that merely having the questionnaire read to does not impact the responses provided by tested subjects, as this is apparently the only way to offer this resource to poorly educated subjects in order to assess the outcomes of the use of hearing aids.

At last, further studies should be conducted to verify the psychometric properties of the IOI-HA using more extensive samples and other test application contexts.
